# Addressing the New Normal in Health Care Using an Interprofessional Model of Care Coordination

**DOI:** 10.1093/geroni/igab046.1259

**Published:** 2021-12-17

**Authors:** Sam Cotton, Anna Faul, Pamela Yankeelov

**Affiliations:** 1 University of Louisville Trager Institute, University of Louisville Trager Institute, Louisville KY, Kentucky, United States; 2 University of Louisville, University of Louisville, Kentucky, United States

## Abstract

There has been significant discourse surrounding the widespread system failures within healthcare during COVID-19. Older, frailer, and poorer persons across the United States have been the most impacted by the pandemic. Given this, our FlourishCare team, received funding through the COVID Cares Act, as part of our Geriatric Workforce Enhancement Program (GWEP) grant, to create innovative programming for individuals that were the most impacted by the pandemic. Remote patient monitoring (RPM) is one intervention been shown as an effective way to assist persons in managing their conditions. Patients from our Optimal Aging Clinic were identified as struggling with hypertension, diabetes and/or COPD. Interprofessional teams of nursing and social work learners were assigned to work with patients. The kits contain all of the necessary technology and a virtual app platform that allows a patient to check their heart rate, blood pressure, oxygen levels, and glucose levels. This information was then disseminated to the team coordinating the patient’s care. The sample was mostly female (88%), African American (64%) and retired (70%). The mean age was 60 (SD=4), and 40% had less than a high school diploma. After 3 months in the program, we saw a significant improvement across all determinants of health, with a particular overall change in access to health services and individual health behaviors. This study showed the importance of providing patients with access to technology and the support of an interprofessional team can improve patient outcomes, lead to improvements in individual health behaviors and improve health literacy.

